# An Observational Study of Multi-Faceted Demyelinating Disorders

**DOI:** 10.7759/cureus.43775

**Published:** 2023-08-19

**Authors:** Nihar R Biswal, Ashok K Mallick, Kali Swain, Jyoti Prakash Sahoo

**Affiliations:** 1 Neurology, Srirama Chandra Bhanja (SCB) Medical College, Cuttack, IND; 2 Pharmacology, Kalinga Institute of Medical Sciences, Bhubaneswar, IND

**Keywords:** multiple sclerosis and other demyelinating disorders, global epidemiology, clinically isolated syndrome, acute transverse myelitis (atm), neuromyelitis optica spectrum disorder (nmosd), demyelinating dieases

## Abstract

Background and objectives: Idiopathic inflammatory demyelinating diseases of the central nervous system (IIDCDs) are wide-ranging disorders due to their similarities and differences. In order to address these conditions, studying their characteristics is essential. The endpoints of our study were to assess the incidence, presenting features, MRI findings, and predictors of disease progression of prevalent demyelinating disorders.

Material and methods: This prospective, observational study was conducted at Srirama Chandra Bhanja (SCB) Medical College and Hospital, India, from August 2018 to November 2021. Individuals of 18-65 years of age with common demyelinating disorders were assessed at baseline, six, 12, and 24 months. Univariate and multivariate analyses were performed for the assessment of predictors. We used R software (version 4.2.1; R Foundation for Statistical Computing, Vienna, Austria) for data analysis.

Results: Two hundred twenty (79%) of 278 enrolled participants completed this study. The mean age of the study population was 52.3±11.4 years. One hundred thirty-eight (63%) of them were males. The most common IIDCD in our study was neuromyelitis optica spectrum disorder (NMOSD: 87, 39.5%), followed by multiple sclerosis (MS: 72, 32.7%), acute transverse myelitis (ATM: 35, 15.9%), and acute disseminated encephalomyelitis (ADEM: 26, 11.8%). The univariate analysis revealed that male gender, diabetes mellitus, and history of smoking or alcoholism were significant predictors of the disease progression.

Conclusion: The IIDCDs were polysymptomatic at the initial presentation. Male diabetics are more prone to progressive disorders. However, multivariate analysis did not provide statistically significant results.

## Introduction

Idiopathic inflammatory demyelinating disorders of the central nervous system (IIDCDs) are a wide range of diverse illnesses that typically have a detrimental effect on people's health on many levels, including their social lives [1]. Lack of a validated differentiation framework based on clinical, radiologic, and pathologic criteria creates uncertainty in the nosology of IIDCDs [2]. In light of the treatments that are available, a prompt diagnosis is essential for reducing relapse and residual disability from such illnesses. IIDCD progression predictors must additionally be evaluated to clarify potential grey areas [3,4].

Age-standardized global prevalence rates for multiple sclerosis (MS) and neuromyelitis optica spectrum disorder (NMOSD) were 7.8 and 2.6 per 100,000 individuals, respectively [5]. Epidemiological knowledge regarding MS and similar demyelinating ailments is scarce in India. The multitude of symptoms, MRI findings, and the severity of disease at initial diagnosis alter the response towards treatment and rapidity of advancement [6,7]. We conducted this study to determine the incidence, clinical features, MRI findings, and predictors of the progression of prevalent demyelinating disorders.

## Materials and methods

This prospective observational study was conducted from August 2018 to November 2021 at SCB Medical College, Cuttack, Odisha, India. We received approval (IEC application no: 036 dated 09.07.2018) from the Institutional Ethics Committee of SCB Medical College before study initiation. Consent from the participants was obtained prior to their enrolment. Adult patients of both sexes diagnosed with either MS as per Macdonald’s criteria [8], NMOSD as per Wingerchuk’s criteria [9], acute transverse myelitis (ATM), or acute disseminated encephalomyelitis (ADEM) according to monophasic and multiphasic forms [10] were included in the study. The persons with infectious etiology, vasculitis, sarcoidosis, systemic autoimmune disease with CNS manifestation, leukodystrophy, and mitochondrial disorders were excluded from the study. The primary objective was to determine the incidence and presenting symptoms. The secondary objectives were to assess the change in MRI findings and the predictors of disease progression at 24 months from baseline.

We used convenience sampling for this study. The 1.5 Tesla scanners were utilized for gadolinium contrast brain and spinal cord imaging. Transfected HEK-2 cells and primate optic nerve served as substrates for immunofluorescence to detect IgG antibodies against aquaporin-4. We implemented convenience sampling for this study. The univariate and multivariate analyses were performed for the predictors of disease progression at 24 months. The variables used for such analyses were age, gender, body weight, body mass index (BMI), history of alcoholism or smoking, congenital brain anomalies, brain infections like encephalitis, meningitis, brain tumors, cerebral palsy, developmental disabilities, head injury, epilepsy, thromboembolic stroke, connective tissue disorders, autism, history of post-traumatic seizures, comorbidities, use of psychoactive substances and the presenting symptoms. The statistical significance level was set at 0.05. For data analysis, we used R software (version 4.2.1; R Foundation for Statistical Computing, Vienna, Austria) [11].

## Results

We screened 320 patients with IIDCD for this study. Twenty-seven patients were beyond the stipulated age range; four had either meningitis or encephalitis, two had vasculitis, one had an autoimmune disorder, and eight did not consent to their participation. The remaining 278 were enrolled. Fifty-eight patients came for only some of the follow-up visits. Finally, 220 participants completed the study and were analyzed. The baseline demographic and clinical variables of the study population are shown in Table 1. The mean age of the study population was 52.3±11.4 years. One hundred thirty-eight (63%) of them were males.

**Table 1:** Baseline sociodemographic and clinical parameters of the study population. The categorical and continuous data were expressed as n (%) and mean ± standard deviation. MS: multiple sclerosis, NMSOD: neuromyelitis optica spectrum disorder, ATM: acute transverse myelitis, ADEM: acute disseminated encephalomyelitis, BMI: body mass index, DM: diabetes mellitus, CNS: central nervous system.

Parameters	Total (n = 220)	MS (n = 72)	NMOSD (n = 87)	ATM (n = 35)	ADEM (n = 26)	p-value
Age (years)	52.3 ± 11.4	46.7 ± 13.8	54.6 ± 17.1	49.2 ± 9.9	57.5 ± 8.0	0.006
Male, n (%)	138 (62.7%)	44 (61.1%)	57 (65.5%)	22 (62.9%)	15 (57.7%)	0.109
Weight (kg)	50.7 ± 14.2	51.6 ± 13.6	47.37 ± 16.4	53.8 ± 11.5	45.0 ± 10.9	0.227
BMI (kg/m^2^)	22.8 ± 2.9	24.1 ± 2.3	23.7 ± 3.4	21.5 ± 2.1	22.2 ± 1.9	0.743
Duration of disease (months)	5.6 ± 5.2	40.7 ± 22.4	4.5 ± 1.3	0.03 ± 0.01	0.12 ± 0.04	< 0.001
Presenting complaints						
Polysymptomatic	163 (74.1%)	49 (68.1%)	66 (75.9%)	25 (71.4%)	23 (88.5%)	0.042
Pyramidal	96 (43.6%)	27 (37.5%)	41 (47.1%)	17 (48.6%)	11 (42.3%)	0.144
Optic	61 (27.7%)	19 (26.4%)	32 (36.8%)	4 (11.4%)	6 (23.1%)	0.183
Sensory	83 (37.7%)	26 (36.1%)	44 (50.6%)	8 (22.9%)	5 (19.2%)	0.127
Urinary	74 (33.6%)	21 (29.2%)	39 (44.8%)	5 (14.3%)	9 (34.6%)	0.195
Cerebellar	27 (12.3%)	8 (11.1%)	11 (12.6%)	3 (8.6%)	5 (19.2%)	0.346
Seizure	16 (7.3%)	2 (2.8%)	5 (5.7%)	2 (5.7%)	7 (26.9%)	< 0.001
Fever	11 (5.0%)	2 (2.8%)	2 (2.3%)	1 (2.9%)	6 (23.1%)	< 0.001
Abnormal MRI findings						
Brain	128 (58.2%)	72 (100%)	24 (27.6%)	6 (17.1%)	26 (100%)	< 0.001
Spinal cord	150 (68.2%)	58 (80.6%)	73 (83.9%)	15 (42.9%)	4 (15.4%)	< 0.001
Comorbidities						
Type 2 DM	117 (53.2%)	31 (43.1%)	63 (72.4%)	14 (40.0%)	9 (34.6%)	< 0.001
Hypertension	59 (26.8%)	14 (19.4%)	34 (39.1%)	6 (17.1%)	5 (19.2%)	0.018
Other cardiovascular disorders	28 (12.7%)	9 (12.5%)	13 (14.9%)	3 (8.6%)	3 (11.5%)	0.215
Epilepsy	37 (16.8%)	14 (19.4%)	17 (19.5%)	4 (11.4%)	2 (7.7%)	0.302
Depression or anxiety	31 (14.1%)	9 (12.5%)	18 (20.7%)	3 (8.6%)	1 (3.8%)	< 0.001
Other CNS disorders	46 (20.9%)	11 (15.3%)	28 (32.2%)	2 (5.7%)	5 (19.2%)	0.014

**Figure 1:**
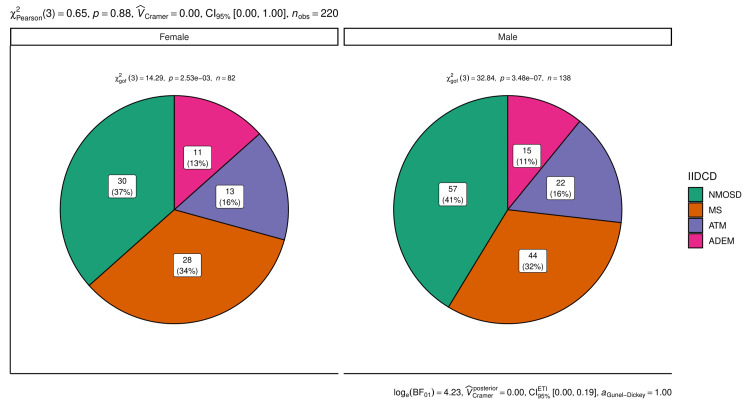
Gender distribution pattern of the diseases. The pie charts show the number (percentage) of females and males affected by idiopathic inflammatory demyelinating disorders of the central nervous system (IIDCDs). MS: multiple sclerosis, NMSOD: neuromyelitis optica spectrum disorder, ATM: acute transverse myelitis, ADEM: acute disseminated encephalomyelitis.

The gender distribution patterns of the disease are shown in Figure 1. The proportion of females and males affected by the idiopathic inflammatory demyelinating disorders of the central nervous system (IIDCDs) were similar (p = 0.88). However, the goodness-of-fit models suggest that intragroup variations were statistically significant (p < 0.001).

**Figure 2:**
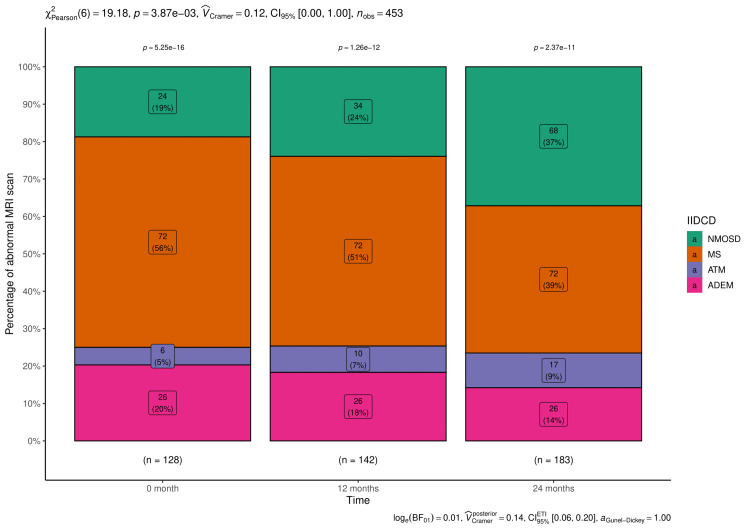
MRI scan results of brain at various time points. The stacked bar diagrams show the number (percentage) of abnormal findings of MRI brain of the participants with the idiopathic inflammatory demyelinating disorders of the central nervous system (IIDCDs). MS: multiple sclerosis, NMSOD: neuromyelitis optica spectrum disorder, ATM: acute transverse myelitis, ADEM: acute disseminated encephalomyelitis.

**Figure 3:**
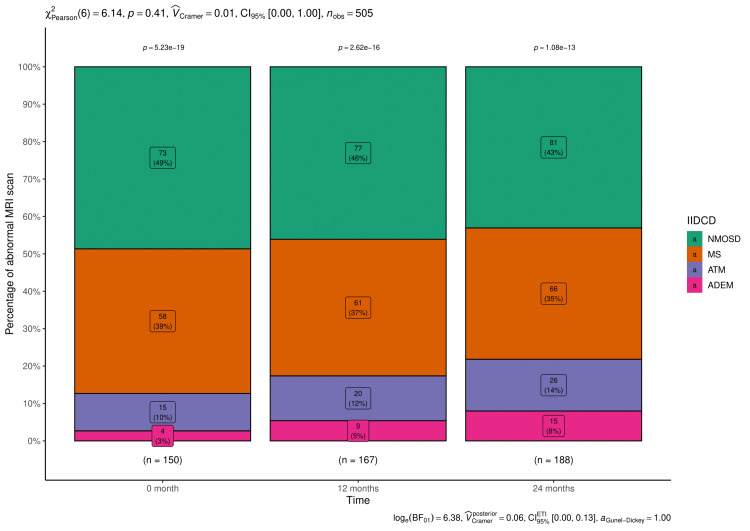
MRI scan results of spinal cord at various time points. The stacked bar diagrams show the number (percentage) of abnormal findings of MRI spine of the participants with idiopathic inflammatory demyelinating disorders of the central nervous system (IIDCDs). MS: multiple sclerosis, NMSOD: neuromyelitis optica spectrum disorder, ATM: acute transverse myelitis, ADEM: acute disseminated encephalomyelitis.

The number and percentages of abnormal MRI brain and spinal cord findings are shown in Figures 2, 3, respectively. The numbers of abnormal MRI brain (p < 0.001) and spine (p = 0.41) reports increased with advanced time.

We investigated the predictors of disease progression at 24 months. Twenty variables including age, gender, body weight, BMI, history of alcoholism, smoking, congenital brain anomalies, brain infections like encephalitis, meningitis, brain tumors, cerebral palsy, developmental disabilities, head injury, epilepsy, thromboembolic stroke, connective tissue disorders, autism, history of post-traumatic seizures, comorbidities, use of psychoactive substances were evaluated as potential predictors of disease progression. We performed univariate and multivariate analyses for the same. The findings are illustrated in Table 2. The disease progression in the participants within 24 months could be attributed to male gender, diabetes mellitus, and history of smoking or alcoholism. However, the multivariate analysis for all the variables was non-significant.

**Table 2:** The predictors of progression of idiopathic inflammatory demyelinating disorders of the central nervous system (IIDCD). All 220 patients with 957 abnormal MRI reports (considering both brain and spine scan at three time points, a total of 1320 patient reports were done) were assessed for the predictors with 20 variables including age, gender, birth weight, body mass index (BMI), history of alcoholism, smoking, congenital brain anomalies, brain infections like encephalitis, meningitis, brain tumors, cerebral palsy, developmental disabilities, head injury, epilepsy, etc. Only variables with p < 0.05 are shown here. Multivariate analysis of all these variables was non-significant.

Attribute	Number of patient reports with attribute	Number of abnormal MRI brain and/or spine in patients with attribute, n (%)	Number of abnormal MRI brain and/or spine in patients without attribute, n (%)	p-value, univariate	Risk ratio (95% CI), univariate
Male gender	828	573 (43.4%)	384 (29.1%)	0.03	1.13 (1.02 – 1.36)
Diabetes mellitus	804	540 (15.5%)	417 (7.1%)	0.008	1.20 (1.09 – 1.42)
History of smoking or alcoholism	696	498 (37.8%)	459 (50.1%)	0.04	1.03 (1.01 – 1.11)

## Discussion

This prospective, observational study focused on the incidence, presenting features, MRI findings, and predictors of disease progression of prevalent demyelinating disorders. The prevalent demyelinating disorders were MS, NMOSD, ATM, and ADEM. All the participants were followed up for 24 months after their enrolment. We found that most participants were polysymptomatic, especially the pyramidal symptoms. The disease durations of the participants were far-flung because of the acute course of ATM and ADEM. The disease progression in the participants could be attributed to male gender, diabetes mellitus, and history of smoking or alcoholism.

The most common IIDCD in our study was NMOSD (87, 39.5%), followed by MS (72, 32.7%), ATM (35, 15.9%), and ADEM (26, 11.8%). The presenting symptoms were pyramidal, optic, sensory, urinary, cerebellar, seizure, and fever. Most patients were polysymptomatic. Our findings were similar to the studies by Mealy et al. [12] and Pandit et al. [13]. The studies by Liu et al. [14] and Viswanathan et al. [15] found that the MRI findings of patients with IIDCDs like MS, NMOSD, and ATM deteriorate with time. Our study findings concord with their findings in this regard. Barhate et al. [16] found that male smokers are more prone to have progressed disorders. Our study findings were consistent with their findings.

This was the first study to evaluate changes in MRI findings and the predictors of the progression of IIDCDs. However, the findings of this study should be analyzed with a few limitations. Firstly, a higher attrition rate, possibly because of the pandemic, reduced the study population. Secondly, the lack of biological markers could have accounted for inconclusive immunological reports on disease progression.

## Conclusions

Our study concludes that the IIDCDs were polysymptomatic at initial presentation. Male diabetics are more prone to progressive disorders. However, multivariate analysis did not provide statistically significant results. We warrant more extensive studies, including the immunological markers as predictors of treatment outcome and disease progression.
